# How does price variance among purchase channels affect consumers’ cognitive process when shopping online?

**DOI:** 10.3389/fpsyg.2022.1035837

**Published:** 2022-11-08

**Authors:** Han Wei, Zhang Xuefeng

**Affiliations:** School of Management, Southwest University of Political Science and Law, Chongqing, China

**Keywords:** price variance, purchase channel, cognitive process, event-related potentials, N2, P3

## Abstract

The rise of a flourishing online shopping market has expanded the range of purchase channels available to consumers. Meanwhile, the competition among channels has become increasingly fierce. In this study, the changes in cognitive processes caused by price variance among channels were investigated using event-related potentials. Several daily necessities with low or high price variance between a self-operated business channel and third-party seller channels were chosen as the study objects from a well-known electronic business platform. Thirty participants’ electroencephalograms were collected while they faced higher or lower price variance during the experiment. The results showed that small price variances between the two channels tended to intensify component N2, while big price variances tended to diminish component P3. These results suggest that N2 may reflect consumers’ identification process for price variance and inhibition of a planned response, while P3 may reflect the activation of attention caused by task difficulty due to price variance. These findings indicate that the changes in ERP components N2 and P3 may act as cognitive indices that measure customers’ identification and attention distribution when considering product price variances among online purchase channels.

## Introduction

With the development of E-commerce, more and more people are turning from traditional purchase channels to online shopping. Online shopping shifts the shopping environment from a real physical environment to a virtual network environment, resulting in substantial differences in shopping characteristics from traditional businesses. The latest developments indicate that the B2C shopping website is in the process of platformization, which means that commodities will not only be sold through the B2C self-operated business channel but also be sold through third-party sellers on the B2C platform (Cao and He, 2016; Cao et al., 2019; Wang and Li, 2020). As more and more purchase channels become available to consumers, channel selection problems emerge.

Putting price variance in the context of different online purchase channels, specifically B2C online shopping platform and third-party sellers on that platform, makes the issue of the impact of price variance on consumers’ cognition more interesting. The commodity prices on different channels may vary widely depending on a variety of factors, such as information costs, competitive pricing on the Internet, and marketing strategies. From the perspective of consumers, their cognition not only derive from price difference, but also different reputation of channels. For example, if you were to buy a pair of ASICS sneakers from Amazon, which channel would you choose: the Amazon self-operated channel or a third-party seller on the Amazon platform? Can one expect that the price difference between online channels will affect consumers as it does for traditional channels?

Researchers have acknowledged that commodity price is one of the critical factors driving consumer choice among sellers (Thaler, 2008; Somervuori and Ravaja, 2013; Stewart et al., 2015; Sohn, 2017) and revealed that the price difference can have a significant impact on consumers’ purchase decisions (Riquelme et al., 2016; Voorveld et al., 2016). Little is known about the effect of price difference among channels on consumers’ cognition. Prior research mainly discussed that consumers could make different decisions based on different channels’ prices (Gino et al., 2017; Gao et al., 2019; Li et al., 2022). Because consumers can indeed perceive differences in price (Wu et al., 2015; Karmarkar et al., 2019), tend to compare the spending between channels and make different choices to get the best deal (Hamilton and Chernev, 2013; Stefano and Noriaki, 2019). For example, consumers will weigh whether it is necessary and how much time and energy would be needed to get the money that could be saved due to the price difference between channels (Fassnacht and Unterhuber, 2016). This indicates that consumers could make different decisions based on different channels’ prices (Gino et al., 2017; Gao et al., 2019; Li et al., 2022). It can be observed that the commodity price and purchase channel are interrelated, and the phenomenon that price differences between channels may affect consumers’ cognitive processes. That is to say, when the same commodity is sold by different entities, the differing prices among sellers may lead to different cognitive processes, which in turn lead to different purchase decisions later.

The methodology the prior research normally uses to test the effect of price differences on consumers’ purchase selection is A/B testing, field experiments, interviews, questionnaires, behavior observation, data analytics within firms, and so on. However, these traditional evaluation methods are not always feasible, because people who participate in surveys are not entirely rational, and consumers may be affected by many other factors such as emotion and context when dealing with information (Solnais et al., 2013; Chneider and Woolgar, 2015; Heather et al., 2019; Bettiga et al., 2020). The traditional methods may not fully reveal the consumer response to marketing stimuli (Sharad and Tanusree, 2015). Whether the study uses a group discussion or an in-person interview, whether the data are confidential or not, consumers’ self-reports are still the main resources to conduct a survey (Hsu, 2017). Some limitations accompany these methods. First, the researchers assume that the respondents can describe the whole process of recognizing problems, analyzing the problems, and making decisions. In fact, many subconscious cognitive processes are not known to the respondents, or cannot be accurately described in words (Olteanu, 2015). Second, other factors such as incentives, time constraints, and peer pressure may also drive respondents to distort their feelings (Erik et al., 2010). As a result, their real thoughts may not be easily reflected by the use of a survey alone. A new approach is needed that can provide a supplement to traditional methods. In this study, we attempt to adopt neuroimaging tools to reveal the cognitive processes that are involved when consumers deal with price differences among online channels. Exploring consumers’ cognitive responses may have the potential to find neuroelectrophysiological evidence to unpack the mechanism linking pricing strategy and consumer behavior, and thus strengthen the evaluation methods based on traditional marketing data.

Scholars have gradually realized the importance of observing consumers’ brain responses to stimuli as a way to understand consumer behavior. With neuroimaging tools such as functional magnetic resonance imaging (fMRI) and brain event-related potentials (ERP), researchers can directly observe consumers’ cognitive processes at the brain level (Association, A. P, 2013, Zhu et al., 2022). The consumer’s brain response to different marketing stimuli can be objectively and quantitatively recorded and analyzed (Clithero, 2018; Meyerding and Mehlhose, 2020; Al-Nabhani et al., 2021). Analyzing these responses not only can supplement traditional research methods, but also make it possible to observe consumer cognitive processes such as pre-judgment, behavioral monitoring, and behavioral prediction, and then provide a more solid theoretical foundation for consumer behavior research. In the current study, we attempt to use ERP to explore the cognitive differences experienced by consumers when facing high or low price variations among channels. The negative ERP waveform that is mainly distributed at the frontocentral areas and evoked during the 250–350 ms time window after stimulus presentation is usually described as N2 (Jonathan and Cyma, 2008). Scholars have suggested that N2 represents the subjects’ identification process, behavioral inhibition process, which is associated with cognitive control processes (Jonathan and Cyma, 2008; Smith et al., 2009; Pandey et al., 2011). The amplitude of N2 is related to the similarity of stimulus materials, with more negative amplitudes for high similarity of stimulus materials than low ones (Nieuwenhuis et al., 2004; Azizian et al., 2006; Kasai et al., 2011; Leek et al., 2016). ERP component P3 is a positive waveform with peak latency around 300–500 ms after the onset of a stimulus (Hagen et al., 2006). P3 is generally considered to be closely related to the attention distribution and target recognition processes (Polich and Comerchero, 2003; Polich, 2007). The amplitude of P3 is generally believed to be related to task difficulty (Polich and Comerchero, 2003; Miller et al., 2011). When the difficulty of the task increases, the attention resources that the subject must devote to the task will increase, and thus the amplitude of P3 will increase, and vice versa (Polich and Corey-bloom, 2005; Hagen et al., 2006).

Based on this, we hypothesize that the interaction of price and channel could modify consumers’ cognitive processes, and that the cognitive change could be reflected in the difference in ERP components N2 and P3 evoked by the high or low price variances between two channels. Specifically, small price variances between channels may tend to evoke a more intense component N2 because a small price variance between channels seems to demand more cognitive resources when subjects perform the task. Meanwhile, smaller price variances between channels may tend to evoke a bigger component P3 because a decrease in price variance between channels may increase the task difficulty and consequently lead to a more intense P3. Our research attempts to make several important contributions to existing literature. Firstly, our research extends and enriches prior research on the relationship of price variance and consumers’ decision, by providing the evidence of the impact of price variance among different online channels on consumers’ cognition. Secondly, our finding that the price-driven differences in the channels’ impacts on consumers at the brain level can not only make up for the shortcomings of traditional research methods, and also strengthen the consumer behavior theory. Thirdly, our work contributes to marketing strategies that help online platforms and third-party sellers to formulate reasonable price competition strategies and enable them to rid itself of meaningless promotional competition.

## Research method

### Subjects

Thirty-three right-handed undergraduate students were recruited for this study. Three subjects were eliminated in the later stages due to excessive EEG artifacts, leaving 30 valid participants remaining for analysis (15 males and 15 females, mean age 24.8 ± 2.6). Normal or corrected-to-normal vision was reported by all 30 participants. None of them had neurological or mental illness, head trauma, or drug abuse, and none were taking medication within 1 month before the experiment. All participants were native Chinese speakers. Written informed consent was obtained from each subject before the experiment, in line with The Code of Ethics of the World Medical Association (Declaration of Helsinki), printed in the British Medical Journal (18 July 1964). The experimental protocol was approved by the local Ethics Committee.

Before the experiment, verbal communication with each participant was conducted to determine frequency of online shopping, spending on online shopping per month, and favorite forms of promotion. The survey showed that all participants were familiar with online shopping. All participants had purchased daily necessities from E-commerce platforms in the past 3 months, and all were aware of the differences between commodities sold or services provided by self-operated channels vs. other third-party seller channels on the platform. After the experiment, a small gift worth about five USD was given to each participant as compensation for participation.

### Stimuli

Two types of online shopping channels were examined in this study: one self-operated business channel vs. other third-party seller channels on one specific platform in China. The commodities involved were all daily necessities such as shampoo, toothpaste, snacks, and washing products, which were selected as experimental materials because they are all closely related to the participants’ lives. All participants were familiar with the selected commodities. According to the market trading rules, commodities sold by the self-operated business channel or the third-party seller are all genuine, excluding counterfeiting and refurbishment. The reasons for the price differences included operating costs, business strategies, services provided, etc. Due to the different online shopping channels for the same commodities, the after-sale guarantees, delivery services, and payment methods available to consumers were also different. Self-operated channels support cash on delivery, fast delivery service (usually <2 days), and unconditional return and replacement (no pickup fee). Third-party sellers do not support cash on delivery service, and the goods transportation service is relatively slow (usually 3–7 days). Moreover, the return service of third-party sellers is more restricted than that of the self-operated channel. For example, in the case of Cetaphil Cleanser mentioned later in this study, consumers who purchase it through the self-operated channels will have it delivered by the self-operated express within 24 h, while those who purchase it from third-party sellers will have to wait at least 3 days for delivery; the self-operated channels also provides no-reason returns and door-to-door return services, while third-party sellers require consumers to negotiate with the sellers before consumers can carry out the more cumbersome return procedures, such as having to send the returned goods themselves. All participants had purchased goods from self-operated and third-party sellers before the experiment, and they were all familiar with the above differences. In addition, the experimenters listed these differences in their verbal communication for the participants.

**Figure 1:**
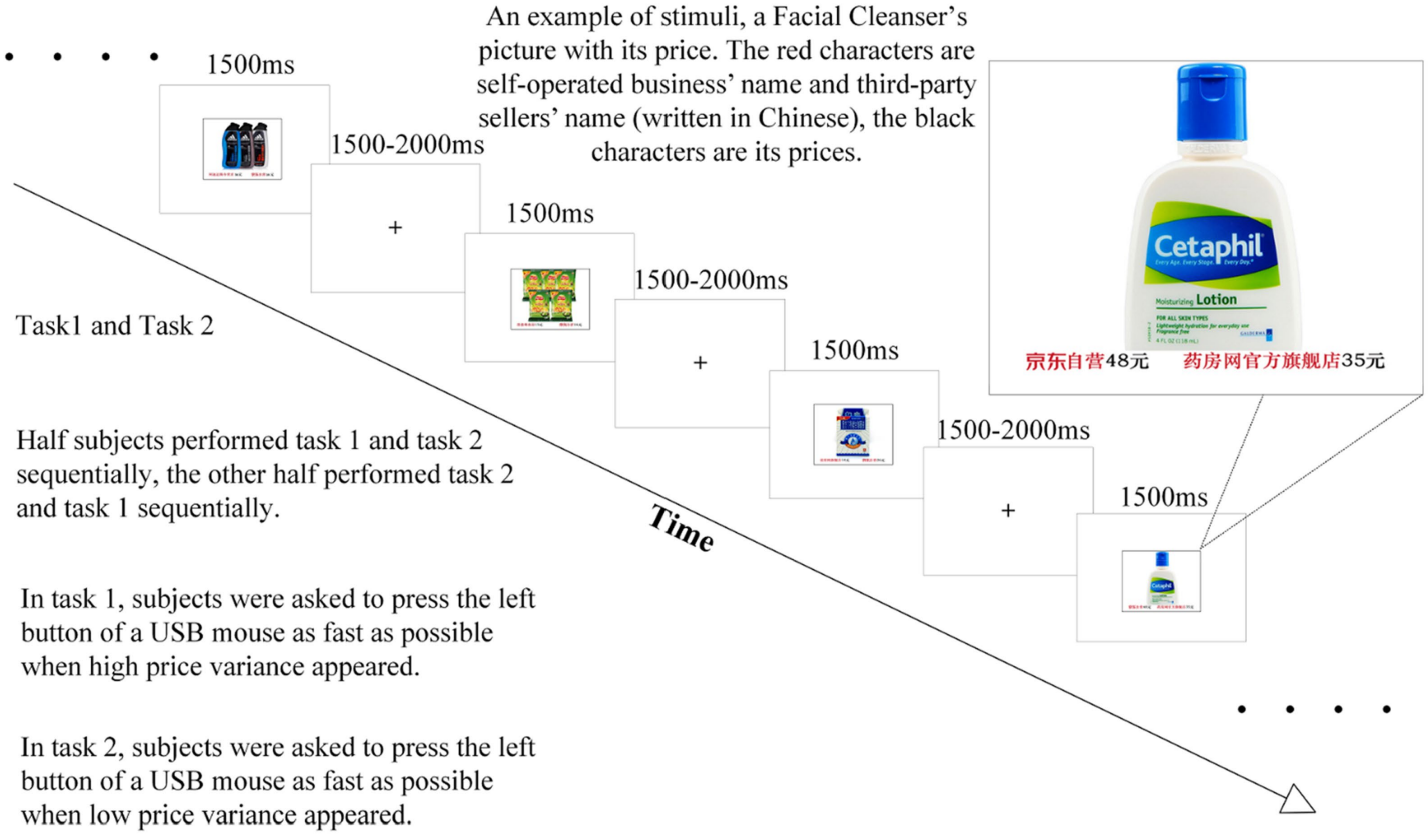
Schematic drawing of the paradigm.

An illustration of the basic stimuli and presentation sequence is given in Figure 1. Twenty-four color photographs of daily necessities with different prices were chosen as critical stimuli. The purchase channels and prices from the self-operated business vs. third-party sellers were shown paired in the right and left bottom corners of the pictures. Following the examples of the market survey and prior studies (Jones et al., 2011, 2012), the price variances between channels on an E-commerce platform were simulated by preset high and low price variances in this study: self-operated business prices were ~25% and 5% higher than those of third-party sellers, half and half. For example, for the situation in which the self-operated channel’s price was about 5% higher than the third-party sellers’ price, we set the price of Cetaphil Cleanser as sold by the self-operated channel to 48 RMB, and set the price as sold by the Yaofang store to 46 RMB (“Yaofang store” is a third-party seller’s name on the platform); while for the situation in which the self-operated channel’s price was about 25% higher than the third-party sellers’ price, the price of Cetaphil Cleanser sold by the self-operated channel was still 48 RMB, but the price as sold by the Yaofang store was 35 RMB. In order to eliminate unnecessary interference factors, the picture did not include spokespersons or product users. Adobe Photoshop was used to ensure that all stimulus pictures had the same brightness and root mean square (RMS) contrast and visual complexity.

### Experimental design

An amended Go/No-go experimental paradigm was used in this experiment. The subjects performed two tasks in total, each of which was composed of 12 pictures in which the self-operated business prices were ~25% than those of the third-party sellers and 12 pictures in which the self-operated business prices were ~5% higher than those of the third-party sellers. In order to exclude the influences on ERPs caused by presentation to the left or right visual field (Woldorff et al., 1997), and to avoid any influences caused by the probability of presentation (Falkenstein et al., 1995), the two different channel prices were randomly presented in the picture’s left or right corner to ensure that the stimulus sequence could appear in a balanced manner. Half of the subjects performed task 1 and task 2 sequentially. In task 1, following the experimental instruction, subjects were asked to press the left button of a USB mouse as quickly as possible when a high price variance appeared. Then, in task 2, subjects were asked to perform the same action when a low price variance appeared. The other half of the subjects carried out the experiment under the same experimental instructions in each task, but the order was changed to task 2 then task 1. If the accuracy were under 95% in any task, the electroencephalogram data would be discarded in the combination process later. Before the formal experiment, the subjects performed an exercise block for about 5 min to make sure they had familiarized themselves with the whole experimental process.

An example of stimuli and the time line of the experiment can be seen in Figure 1. All stimuli were presented using E-prime (version 2.0 professional) in the center of a gray background LCD screen. Each trial was presented 10 times. Each trial consisted of the presentation of a stimulus (duration of 1,500 ms) followed by a fixation cross to avoid the repeated presentations of the stimuli. The inter-trial interval was random with a duration between 1,500 and 2,000 ms. All trials were presented sequentially in a randomized order. The subjects viewed the stimuli from a distance of 100 cm at the center, with a horizontal visual angle of 10.3° and a vertical visual angle of 6.8°. An electrically shielded and sound-attenuated experimental chamber was used. The participants were seated in a comfortable chair during the experiment. Subjects were offered a rest break for 5 min between tasks.

### Electroencephalogram recording and analysis

The electroencephalogram was collected using the Neuroscan EEG system (Neurosoft Labs Inc) with a band-pass of 0.01–100 Hz and a sample rate of 500 Hz. The acquisition process was continuously recorded and analyzed offline. Ag/AgCl electrodes were mounted in a cap according to the international 10/20 system and located at 34 standard positions (FP1/2, FPZ, F3/4, F7/8, FZ, FC3/4, FT7/8, FCZ, C3/4, T7/8, CZ, CP3/4, TP7/8, CPZ, P3/4, P7/8, PZ, PO3/4, POZ, O1/2, OZ). A reference electrode was placed on the left mastoid and referenced to link mastoids offline. Vertical eye movements were monitored using a vertical electrooculogram that was recorded from the right eye by supra-orbital and infra-orbital electrodes (vertical EOG). Horizontal eye movements were monitored using a horizontal electrooculogram recorded by electrodes on the outer canthi of both eyes (horizontal EOG). The impedance of each electrode was kept below 5 KΩ during the acquisition process. Offline data were processed using Curry7.0 SBA (Neurosoft Labs Inc). Large artifacts caused by muscle or eye movements were manually removed. The trials in which base-to-peak electrooculogram (EOG) amplitude exceeded 200 μV, amplifier saturation occurred, or the baseline shift exceeded 250 μV/s were automatically rejected offline (7%). After band-pass filtering at 0.05–30 Hz (24 dB/Octave), the EEG was epoched offline into 1,000 ms: from 200 ms before picture onset to 800 ms after onset (baseline = 200 ms). The epoched EEG data were later combined to yield two primary conditions: self-operated business prices 5% higher vs. 25% higher than third-party sellers.

## Results

**Figure 2:**
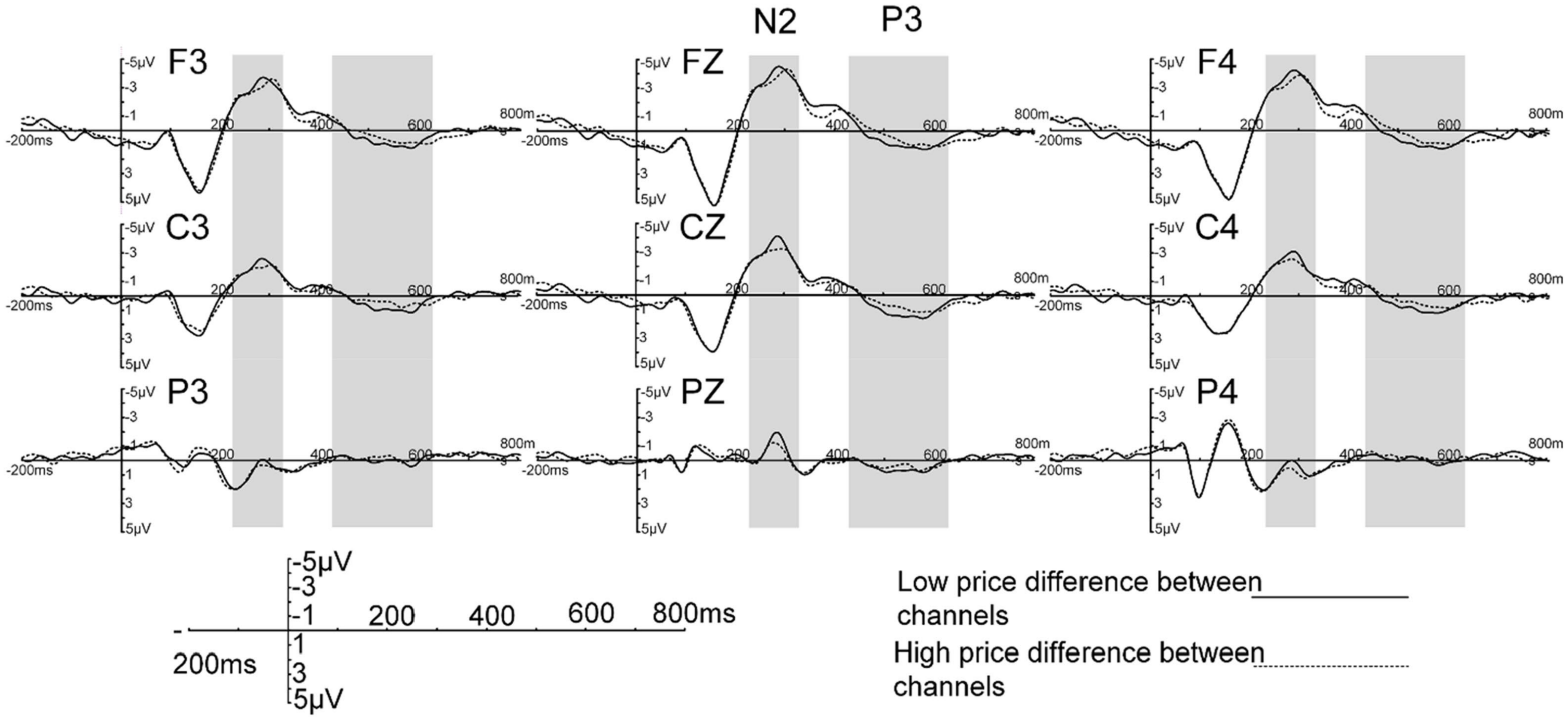
Raw ERPs waveforms at nine electrode sites.

**Figure 3:**
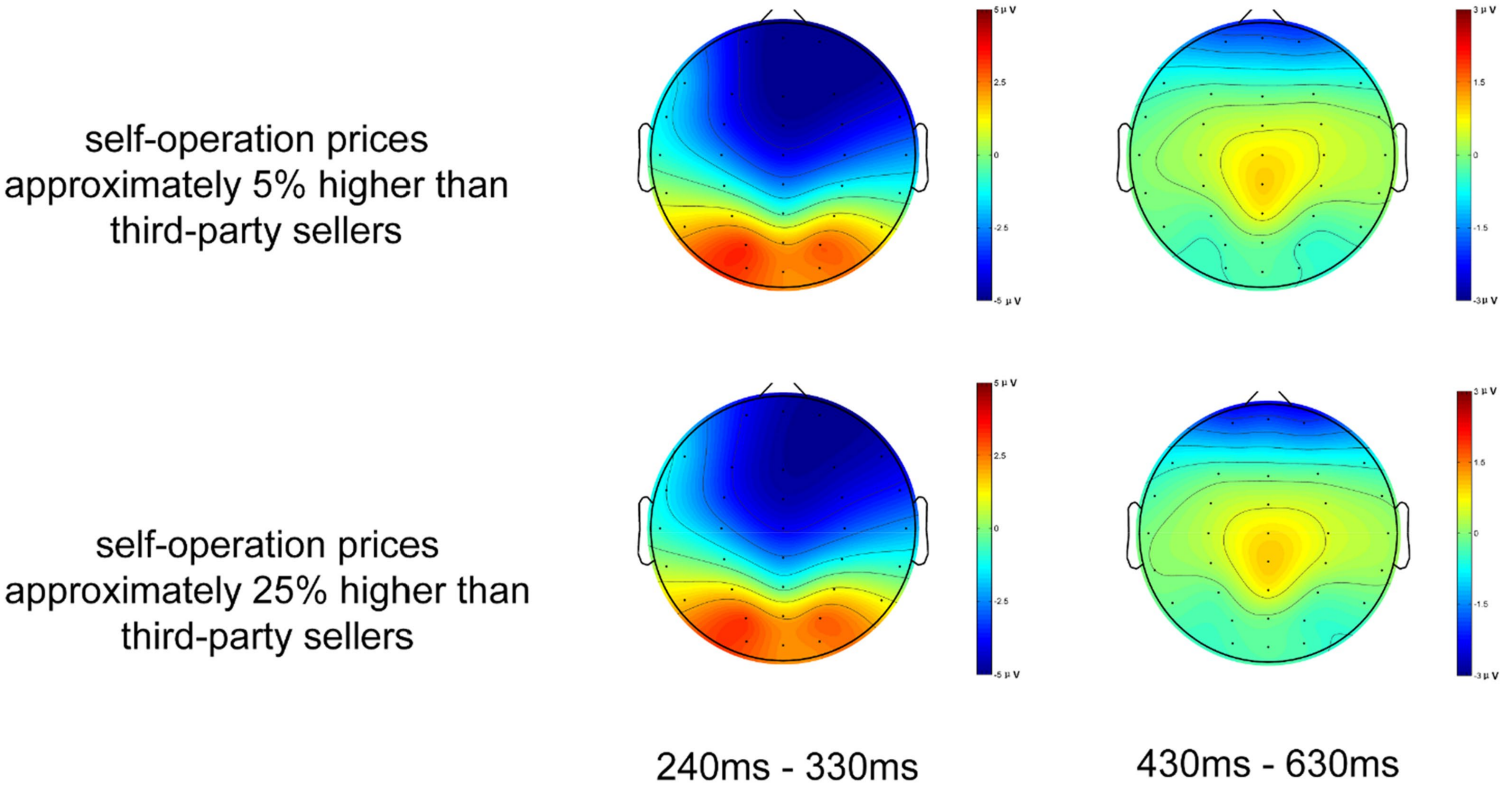
Topographic maps of N2 (240–330 ms) and P3 (430–630 ms).

The raw waveform is presented in Figure 2. Averaged ERP were drawn by Curry7.0 SBA (Neurosoft Labs Inc). Based on a visual examination of the potential distributions and the scalp topographical mapping of potentials (Figure 3), following prior studies (Eiichi and Yukihiko, 1992; Falkenstein et al., 1999; Jonathan and Cyma, 2008), the mean amplitudes of N2 within a 240–330 ms time window at the nine electrodes F3, FZ, F4, C3, CZ, C4, P3, PZ, P4 were selected for analysis. Similarly, the mean amplitudes of P3 within a 430–630 ms time window at the nine electrodes F3, FZ, F4, C3, CZ, C4, P3, PZ, P4 were chosen for analysis, following the example of previous studies (Wiese and Schweinberger, 2011; Dinteren et al., 2014; Shang et al., 2016). A within-subjects repeated measures ANOVA was used to compare the mean amplitudes of N2 and P3, with primary conditions (self-operated business prices ~25% higher vs. self-operated business prices ~5% higher) and distribution as two within-subject factors. The basic descriptive statistics of evoked N2 and P3 potentials can be seen in Table 1. For all statistical effects involving two or more degrees of freedom in the numerator, the Greenhouse–Geisser epsilon was used to correct possible violations of the sphericity assumption when appropriate. The significance level was set at *p* < 0.05.

**Table 1:** The basic descriptive statistics of evoked.

Distribution		25% higher	5% higher	*t*-Value	Value of *p*
F3	N2	−3.23 ± 1.95	−3.84 ± 2.15	−3.841	0.001
	P3	−0.64 ± 0.94	−0.19 ± 1.10	2.632	0.013
Fz	N2	−4.29 ± 2.33	−4.87 ± 2.25	−2.599	0.015
	P3	−0.56 ± 0.94	−0.12 ± 1.02	2.872	0.008
F4	N2	−4.18 ± 1.79	−4.81 ± 2.16	−2.840	0.008
	P3	−0.53 ± 1.04	−0.13 ± 1.02	2.509	0.018
C3	N2	−1.89 ± 1.91	−2.35 ± 1.79	−2.910	0.007
	P3	−0.07 ± 0.88	0.26 ± 0.95	2.516	0.018
Cz	N2	−3.21 ± 1.97	−3.90 ± 1.97	−3.840	0.001
	P3	0.20 ± 0.93	0.58 ± 0.88	2.675	0.012
C4	N2	−2.57 ± 1.87	−3.11 ± 1.58	−3.141	0.004
	P3	0.00 ± 0.83	0.30 ± 0.81	2.641	0.013
P3	N2	1.51 ± 1.73	1.21 ± 1.60	−2.442	0.021
	P3	−0.25 ± 0.74	−0.05 ± 0.72	2.368	0.025
Pz	N2	0.01 ± 1.63	−0.517 ± 1.44	−3.353	0.002
	P3	0.21 ± 1.08	0.47 ± 0.96	2.586	0.015
P4	N2	1.35 ± 1.56	1.01 ± 1.39	−2.413	0.022
	P3	−0.33 ± 0.92	−0.07 ± 0.80	3.043	0.005

The results showed that price variances caused significant differences in the component N2 ([*F* (1, 29) = 10.818, *p* = 0.003]) and in distribution ([*F* (8, 232) = 173.840, *p* < 0.001]), but not in the price variance × distribution interaction ([*F* (8, 232) = 1.788, *p* = 0.166]). Combining the raw waveform and the scalp topographical mapping with variance analysis, the results demonstrated that high price variance conditions were associated with smaller N2 amplitudes than were low price variance conditions. Stimuli with low price variance elicited a more negative N2 than those with high price variance, which were distributed broadly over the scalp and maximal on the fronto-central scalp.

The price variance also caused significant differences in the component P3 ([*F* (1, 29) = 5.980, *p* = 0.021]) and in the distribution ([*F* (8, 232) = 10.441, *p* < 0.001]), but not in the price variance × distribution interaction ([*F* (8, 232) = 1.783, *p* = 0.165]). We also found that the average P3 amplitude was larger for low price variance conditions than for high price variance conditions. Stimuli with low price variance elicited a more positive P3 than did those with high price variance, and the P3 was also distributed broadly over the whole surface of the scalp except the left temporal and parietal-occipital scalp.

## Discussion

The results showed that, in the time window of 240–330 ms, there were significant differences in the N2 potentials evoked by high vs. low price variance between channels. A high price variance between channels resulted in a smaller N2, which was mainly located in the fronto-central area, as seen in Figures 2, 3. It has been assumed that N2 distributed over the front-central area of the scalp reflects the process of cognitive control (Luck and Kappenman, 2012; Susana et al., 2014). The average amplitude is related to the similarity between the stimulus materials: the higher the degree of similarity, the larger the N2 (Nieuwenhuis et al., 2004; Azizian et al., 2006). We posit, based on previous studies, that the component N2 reflects participants’ identification of price variance information (Salil and Pierre, 2005; Hu et al., 2013; Wiese et al., 2014). Stimulation with price variance information has been divided into two categories in this experiment, high and low. When the stimulus appeared, subjects had to distinguish task stimuli according to the experiment introduction with the smallest delay possible. We believe that the change in N2 represents the cognitive resources required when identifying the price variances. A small difference between the prices for the two channels costs more resources to identify, while participants could relatively easily identify a high price variance, in which the differences between prices is a little significant. As N2 potentials evoked by stimuli with price variance show, the average amplitude decreased when high price variance appeared, which is consistent with prior findings (Schweinberger et al., 2002; Pawel et al., 2011). Another interpretation is that N2 may reflect the inhibition of a planned response. As some scholars have pointed out in prior research (Pfefferbaum et al., 1985; Bruin and Wijers, 2002), N2 tends to be larger in tasks for which an overt response must be withheld in the Go/No-go paradigm. The No-go stimuli shared most features with the Go stimuli and differed only in price variance. Thus, the preparation of an incorrect response that must be suppressed was triggered while participants proceeded in their task (Jonathan and Cyma, 2008). The overall similarity in the small price variances resulted in larger N2 when participants were exposed to a relatively difficult trial, which means that low price variance stimuli elicited larger N2 than high price variance stimuli. This speculation is consistent with a prior study that found that difficult No-go trials elicited larger N2 than easy No-go trials (Nieuwenhuis et al., 2004).

The P3 component embodies similar functional connections; the P3 induced by low price variance was greater than that induced by high price variance between channels. The scalp distributions of P3 appeared to be central; therefore, the P3 component might be a P3a-like potential. In one interpretation, the P3, which is distributed over the central area, is thought to reflect the distribution process of attention, the amplitude of P3 component was related to the attention resource devoted (Polich and Comerchero, 2003; Hagen et al., 2006; John and Criado, 2006). We believed that subjects devoted more attention resources to distinguish the 5% condition stimulus materials. As mentioned in section 2.2 above, the differences between the two sets of stimulus materials — i.e. the self-operated channel’s price being either 5% higher or 25% higher than the third-party sellers’ price — would only be observed in the price of 46 RMB or 35 RMB offered by the third-party seller, while the self-operated channel price was still set to 48 RMB for both conditions. When the stimuli were presented, participants allocated fewer attention resources to identify the 25% higher price variance stimuli because 48 and 35 share fewer features than 48 and 46. In other words, the difficulty increased when participants identified the 5% condition stimulus materials, resulting in a more positive P3 component compared to the 25% higher price condition because more attention resource need to be devoted to identify the harder stimuli (Hagen et al., 2006). Another interpretation is that P3a may be subtended by neural changes in the anterior cingulate function when new stimuli replace the contents of working memory (Polich and Comerchero, 2003; Khan et al., 2020). According to this interpretation, as participants basically know the average price variance between the self-operated business channel and third-party seller channel in the real world, the stimuli that have a lower price variance could be regarded, in a sense, as a type of non-target distractor. Compared to stimuli that had high price variances between channels, stimuli with low price variances would cause participants to be more risk-avoidant when considering the possibility of errors and thus subsequently more likely to activate an anterior cingulate/medial prefrontal network during decision-making, thus leading to a greater P3 component. Other researchers have also argued that it is the P3, but not the N2, that is associated with response inhibition or with an evaluation/decision process with regard to the expected and/or given response (Bruin et al., 2001; Smith et al., 2007; Singh and Basu, 2009).

This study has some important differences from traditional studies. Prior research has mainly focused on the effects of multiple prices in traditional distribution channels and the influence of price changes on consumers’ selection. In this study, the interaction of price and channel was investigated by using an experimental approach, and the consumers’ cognitive processes while facing purchase channels with price variances were studied. Neuroscientific evidence was expected to be found to make up for the deficiencies in traditional research to date. Through the observation of consumers’ cognitive reactions to distribution channels with price variances, neuroelectrophysiological indicators of the cognitive processes were preliminarily explored. It was found that some mature ERP indicators in cognitive neuroscience may help to explain the mechanisms of consumer behavior.

## Conclusion

To summarize, the cognitive differences caused by price variance between channels were investigated using event-related potentials in this study. Some daily necessities with high and low price variance between a self-operated business channel and third-party seller channel were presented as experimental materials. The ERP data demonstrated that low price variances between channels induced an intensified N2 and P3 at the fronto-central areas and central areas, respectively. We believe that the different price variances between channels led to the differences in cognitive processes. The amplitude variation in N2 and P3 reflected differences in the identification and attention distribution processes caused by price variances. It can be concluded that EPR components N2 and P3 could serve as a cognitive index to measure consumers’ identification and attention distribution to price variances between purchase channels. This study contributes to our understanding of consumers’ neural activity when facing purchase channel problems with price variances. Exploration of this cognitive process can help companies to set a more reasonable price to participate in market competition and even develop a more effective marketing strategy.

## Implications

Customers’ price perception has a decisive influence on their channel selection decision. The impact of the interaction of price and channel on consumers’ cognitive processes should be taken seriously. Whether a channel’s price is able to attract consumer attention has a significant impact on the purchase decision, and can even indirectly affect product sales and profits. Different channels have different characteristics. Managers should carefully consider the nature of a channel when making its channel pricing strategy and then improve the service level in a targeted way to enhance price competitiveness. This study was not only dedicated to investigating consumers’ cognitive differences caused by price variance between channels but also to providing a new method for analyzing consumers’ cognitive differences caused by the interaction of price and channel. This study has shown that when consumers face the purchasing channel selection problem with price differences, their early cognitive processing stages can be observed using neuroimaging tools. Continued in-depth development of this method can not only help us understand the channel selection process for daily necessities, but is also expected to extend the research field to specialty or luxury markets so that we can acquire a more profound theoretical explanation of universal consumer behavior at the neuroscience level.

## Data availability statement

The raw data supporting the conclusions of this article will be made available by the authors, without undue reservation.

## Ethics statement

The studies involving human participants were reviewed and approved by SWUPL Ethics Committee. The patients/participants provided their written informed consent to participate in this study.

## Author contributions

HW and ZXF: conceptualization and validation. ZXF: methodology, software, formal analysis, writing—original draft preparation, data curation, and visualization. HW: investigation, resources, writing—review and editing, supervision, project administration, and funding acquisition. All authors have read and agreed to the published version of the manuscript.

## Funding

This work was supported by the National Natural Science Foundation of China under Grant (numbers 72032007 and 71972159); the Chongqing Municipal Natural Science Foundation under Grant (number cstc2020jcyj–msxmX1015); and the Chongqing Humanities and Social Science Foundation under Grant (number 22SKGH042).

## Conflict of interest

The authors declare that the research was conducted in the absence of any commercial or financial relationships that could be construed as a potential conflict of interest.

## Publisher’s note

All claims expressed in this article are solely those of the authors and do not necessarily represent those of their affiliated organizations, or those of the publisher, the editors and the reviewers. Any product that may be evaluated in this article, or claim that may be made by its manufacturer, is not guaranteed or endorsed by the publisher.

## References

[ref1] Al-Nabhani K. Wilson A. Mclean G. (2021). Examining consumers’ continuous usage of multichannel retailers' mobile applications. Psychol. Mark. 39, 168–195. doi: 10.1002/mar.21585

[ref2] Association, A. P. (2013). Publication Manual of the American Psychological Association: 6th ed., Washington, DC, American Psychological Association.

[ref3] Azizian A. Freitas A. L. Parvaz M. A. Squires N. K. (2006). Beware misleading cues: perceptual similarity modulates the N2/P3 complex. Psychophysiology 43, 253–260. doi: 10.1111/j.1469-8986.2006.00409.x, PMID: 16805863

[ref4] Bettiga D. Bianchi A. M. Lamberti L. Noci G. (2020). Consumers emotional responses to functional and hedonic products: a neuroscience research. Front. Psychol. 11, 559779. doi: 10.3389/fpsyg.2020.559779 33123043 PMC7573359

[ref5] Bruin K. J. Wijers A. A. (2002). Inhibition, response mode, and stimulus probability: a comparative event-related potential study. Clin. Neurophysiol. 113, 1172–1182. doi: 10.1016/S1388-2457(02)00141-4, PMID: 12088714

[ref6] Bruin K. J. Wijers A. A. Staveren A. S. J. V. (2001). Response priming in a Go/NoGo task: do we have to explain the Go/NoGo N2 effect in terms of response activation instead of inhibition? Clin. Neurophysiol. 112, 1660–1671. doi: 10.1016/S1388-2457(01)00601-0, PMID: 11514249

[ref7] Cao K. He P. (2016). The competition between B2C platform and third-party seller considering sales effort. Kybernetes 45, 1084–1108. doi: 10.1108/K-01-2016-0009

[ref8] Cao K. Xu X. Bian Y. Sun Y. (2019). Optimal trade-in strategy of business-to-consumer platform with dual-format retailing model. Omega 82, 181–192. doi: 10.1016/j.omega.2018.01.004

[ref9] Chneider T. S. Woolgar S. (2015). Neuromarketing in the making: enactment and reflexive entanglement in an emerging field. BioSocieties 10, 400–421. doi: 10.1057/biosoc.2015.37

[ref10] Clithero J. A. (2018). Response times in economics: looking through the lens of sequential sampling models. J. Econ. Psychol. 69, 61–86. doi: 10.1016/j.joep.2018.09.008

[ref11] Dinteren R. V. Arns M. Jongsma M. L. A. Kessels R. P. C. (2014). P300 development across the lifespan: a systematic review and meta-analysis. PLoS One 9:E87347. doi: 10.1371/journal.pone.0087347, PMID: 24551055 PMC3923761

[ref12] Eiichi J. Yukihiko K. (1992). Relation of a negative ERP component to response inhibition in a Go/No-Go task. Electroencephalogr. Clin. Neurophysiol. 82, 477–482. 1375556 10.1016/0013-4694(92)90054-l

[ref13] Erik F. C. Lisa C. Robert K. (2010). Defining neuromarketing: practices and professional challenges. Harv. Rev. Psychiatry 18, 230–237. doi: 10.3109/10673229.2010.496623 20597593 PMC3152487

[ref14] Falkenstein M. Hoormann J. Hohnsbein J. (1999). ERP components in Go/NoGo tasks and their relation to inhibition. Acta Psychol. 101, 267–291. doi: 10.1016/S0001-6918(99)00008-6, PMID: 10344188

[ref15] Falkenstein M. Koshlykova N. A. Kiroj V. N. Hoormann J. Hohnsbein J. (1995). Late ERP components in visual and auditory Go/NoGo tasks. Electroencephalogr. Clin. Neurophysiol. 96, 36–43. doi: 10.1016/0013-4694(94)00182-K, PMID: 7530187

[ref16] Fassnacht M. Unterhuber S. (2016). Consumer response to online/offline price differentiation. J. Retail. Consum. Serv. 28, 137–148. doi: 10.1016/j.jretconser.2015.09.005

[ref17] Gao L. Melero I. Javier S. F. (2019). Multichannel integration along the customer journey: a systematic review and research agenda. Serv. Ind. J. 9, 1–32. doi: 10.1080/02642069.2019.1652600

[ref18] Gino M. Marco M. Sara P. Monica R. Elena T. (2017). “Logistics in Omni-Channel retailing: Modelling and analysis of three distribution configurations,” in *IEEE International Conference On Service Operations And Logistics, And Informatics (Soli)*. Bari, Italy: IEEE.

[ref19] Hagen G. F. Gatherwright J. R. Lopez B. A. Polich J. (2006). P3a from visual stimuli: task difficulty effects. Int. J. Psychophysiol. 59, 8–14. doi: 10.1016/j.ijpsycho.2005.08.003, PMID: 16253363

[ref20] Hamilton R. Chernev A. (2013). Low prices are just the beginning: price image in retail management. J. Mark. 77, 1–20. doi: 10.1080/02642069.2019.1652600

[ref21] Heather A. Helen H. Shaun S. (2019). Using neuroscience to understand the impact of premium digital out-of-home media. Int. J. Mark. Res. 61, 588–600. doi: 10.1177/1470785319851316

[ref22] Hsu M. (2017). Neuromarketing: inside the mind of the consumer. Calif. Manag. Rev. 59, 5–22. doi: 10.1177/0008125617720208

[ref23] Hu X. Pornpattananangkul N. Rosenfeld J. P. (2013). N200 and P300 as orthogonal and integrable indicators of distinct awareness and recognition processes in memory detection. Psychophysiology 50, 454–464. doi: 10.1111/psyp.12018, PMID: 23317115

[ref24] John P. Criado R. J. (2006). Neuropsychology and neuropharmacology of P3a and P3b. Int. J. Psychophysiol. 60, 172–185. doi: 10.1016/j.ijpsycho.2005.12.012 16510201

[ref25] Jonathan R. F. Cyma V. P. (2008). Influence of cognitive control and mismatch on the N2 component of the ERP: a review. Psychophysiology 45, 152–170. doi: 10.1111/j.1469-8986.2007.00602.x 17850238 PMC2365910

[ref26] Jones W. J. Childers T. L. Jiang Y. (2011). “The shopping brain: neural correlates of buying under different promotional formats,” in *Society For Consumer Psychology, Atlanta, GA*. *Vol. 1*. 1–28.

[ref27] Jones W. J. Childers T. L. Jiang Y. (2012). The shopping brain: math anxiety modulates brain responses to buying decisions. Biol. Psychol. 89, 201–213. doi: 10.1016/j.biopsycho.2011.10.011, PMID: 22027087

[ref28] Karmarkar U. R. Shiv B. Knutson B. (2019). Cost conscious? The neural and behavioral impact of price primacy on decision making. J. Mark. Res. 52, 467–481. doi: 10.1509/jmr.13.0488

[ref29] Kasai T. Moriya H. Hirano S. (2011). Are objects the same as groups? ERP correlates of spatial Attentional guidance by irrelevant feature similarity. Brain Res. 1399, 49–58. doi: 10.1016/j.brainres.2011.05.016, PMID: 21652032

[ref30] Khan S. Fazili A. I. Bashir I. (2020). Counterfeit luxury consumption: a review and research agenda. J. Consum. Behav 20, 337–367. Early Access: September 2020. doi: 10.1002/cb.1868

[ref31] Leek E. C. Roberts M. Oliver Z. J. Cristino F. Pegna A. J. (2016). Early differential sensitivity of evoked-potentials to local and global shape during the perception of three-dimensional objects. Neuropsychologia 89, 495–509. doi: 10.1016/j.neuropsychologia.2016.07.006, PMID: 27396674

[ref32] Li J. Guo F. Xu J. Yu Z. (2022). What influences consumers' intention to purchase innovative products: evidence from China. Front. Psychol. 13:838244. doi: 10.3389/fpsyg.2022.838244, PMID: 35432119 PMC9005750

[ref33] Luck S. J. Kappenman E. S. (2012). The Oxford Handbook of Event-Related Potential Components, Oxford New York, Oxford University Press.

[ref34] Meyerding S. G. H. Mehlhose C. M. (2020). Can Neuromarketing add value to the traditional marketing research? An exemplary experiment with functional near-infrared spectroscopy (fNIRS). J. Bus. Res. 107, 172–185. doi: 10.1016/j.jbusres.2018.10.052

[ref35] Miller M. W. Rietschel J. C. Mcdonald C. G. Hatfield B. D. (2011). A novel approach to the physiological measurement of mental workload. Int. J. Psychophysiol. 80, 75–78. doi: 10.1016/j.ijpsycho.2011.02.003, PMID: 21320552

[ref36] Nieuwenhuis S. Yeung N. Cohen J. D. (2004). Stimulus modality, perceptual overlap, and the Go/No-Go N2. Psychophysiology 41, 157–160. doi: 10.1046/j.1469-8986.2003.00128.x, PMID: 14693011

[ref37] Olteanu M. D. B. (2015). Neuroethics and responsibility in conducting neuromarketing research. Neuroethics 8, 191–202. doi: 10.1007/s12152-014-9227-y

[ref38] Pandey A. K. Kamarajan C. Tang Y. Chorlian D. B. Roopesh B. N. Manz N.. (2011). Neurocognitive deficits in male alcoholics: an ERP/sLORETA analysis of the N2 component in an equal probability Go/NoGo task. Biol. Psychol. 89, 170–182. doi: 10.1016/j.biopsycho.2011.10.009 22024409 PMC3245806

[ref39] Pawel T. Katarzyna J. Artur M. Anna N. (2011). How multiple repetitions influence the processing of self-, famous and unknown names and faces: an ERP study. Int. J. Psychophysiol. 79, 219–230. doi: 10.1016/j.ijpsycho.2010.10.010 21035509

[ref40] Pfefferbaum A. Ford J. M. Weller B. J. Skopell B. (1985). Erps to response production and inhibition. Electroencephalogr. Clin. Neurophysiol. 60, 423–434. doi: 10.1016/0013-4694(85)91017-X 2580694

[ref41] Polich J. (2007). Updating P300: an integrative theory of P3a and P3b. Clin. Neurophysiol. 118, 2128–2148. doi: 10.1016/j.clinph.2007.04.019, PMID: 17573239 PMC2715154

[ref42] Polich J. Comerchero M. D. (2003). P3a from visual stimuli: typicality, task, and topography. Brain Topogr. 15, 141–152. doi: 10.1023/A:1022637732495 12705810

[ref43] Polich J. Corey-Bloom J. (2005). Alzheimers disease and P300: review and evaluation of task and modality. Curr. Alzheimer Res. 2, 515–525. doi: 10.2174/156720505774932214, PMID: 16375655

[ref44] Riquelme I. P. Roman S. Iacobucci D. (2016). Consumers' perceptions of online and offline retailer deception: a moderated mediation analysis. J. Interact. Mark. 35, 16–26. doi: 10.1016/j.intmar.2016.01.002

[ref45] Salil H. P. Pierre N. A. (2005). Characterization of N200 and P300: selected studies of the event-related potential. Int. J. Med. Sci. 2, 147–154. doi: 10.7150/ijms.2.147 16239953 PMC1252727

[ref46] Schweinberger S. R. Pickering E. C. Jentzsch I. Burton A. M. Kaufmann J. M. (2002). Event-related brain potential evidence for a response of inferior temporal cortex to familiar face repetitions. Cogn. Brain Res. 14, 398–409. doi: 10.1016/S0926-6410(02)00142-8, PMID: 12421663

[ref47] Shang Q. Huang Y. Ma Q. (2016). Hazard levels of warning signal words modulate the inhibition of return effect: evidence from the event-related potential P300. Exp. Brain Res. 234:1785. doi: 10.1007/s00221-016-4619-3, PMID: 27017601

[ref48] Sharad A. Tanusree D. (2015). Neuromarketing and consumer neuroscience: current understanding and the way forward. Decision 42, 457–462. doi: 10.1007/s40622-015-0113-1

[ref49] Singh S. M. Basu D. (2009). The P300 event-related potential and its possible role as an endophenotype for studying substance use disorders: a review. Addict. Biol. 14, 298–309. doi: 10.1111/j.1369-1600.2008.00124.x, PMID: 18811679

[ref50] Smith J. L. Johnstone S. J. Barry R. J. (2007). Response priming in the Go/NoGo task: the N2 reflects neither inhibition nor conflict. Clin. Neurophysiol. 118, 343–355. doi: 10.1016/j.clinph.2006.09.027, PMID: 17140848

[ref51] Smith J. L. Smith E. A. Provost A. L. Heathcote A. (2009). Sequence effects support the conflict theory of N2 and P3 in the Go/NoGo task. Int. J. Psychophysiol. 75, 217–226. doi: 10.1016/j.ijpsycho.2009.11.002 19951723

[ref52] Sohn S. (2017). Consumer processing of mobile online stores: sources and effects of processing fluency. J. Retail. Consum. Serv. 36, 137–147. doi: 10.1016/j.jretconser.2017.01.008

[ref53] Solnais C. Andreu J. Sánchez-Fernández J. Andréu-Abela J. (2013). The contribution of neuroscience to consumer research: a conceptual framework and empirical review. J. Econ. Psychol. 36, 68–81. doi: 10.6084/M9.FIGSHARE.1504027

[ref54] Somervuori O. Ravaja N. (2013). Purchase behavior and psychophysiological responses to different price levels. Psychol. Mark. 30, 479–489. doi: 10.1002/mar.20621

[ref55] Stefano C. Noriaki M. (2019). Competition between offline and online retailers with heterogeneous customers. Rev. Ind. Organ. 10, 1–18. doi: 10.1007/s11151-019-09734-1

[ref56] Stewart N. Reimers S. Harris A. J. L. (2015). On the origin of utility, weighting, and discounting functions: how they get their shapes and how to change their shapes. Manag. Sci. 61, 687–705. doi: 10.1287/mnsc.2013.1853

[ref57] Susana C.-F. Mónica L. Fernando D. (2014). Effects of amnestic mild cognitive impairment on N2 and P3 Go/NoGo ERP components. J. Alzheimers Dis. 38, 295–306. doi: 10.3233/JAD-130677 23963292

[ref58] Thaler R. (2008). Mental accounting and consumer choice. Mark. Sci. 4, 199–214. doi: 10.1287/mksc.1070.0330

[ref59] Voorveld H. A. M. Smit E. G. Neijens P. C. Bronne A. E. (2016). Consumers' cross-channel use in online and offline purchases. J. Advert. Res. 56, 385–400. doi: 10.2501/JAR-2016-044

[ref60] Wang W. Li F. (2020). What determines online transaction Price dispersion? Evidence from the largest online platform in China. Electron. Commer. Res. Appl. 42:100968. doi: 10.1016/j.elerap.2020.100968

[ref61] Wiese H. Altmann C. S. Schweinberger S. R. (2014). Effects of attractiveness on face memory separated from distinctiveness: evidence from event-related brain potentials. Neuropsychologia 56, 26–36. doi: 10.1016/j.neuropsychologia.2013.12.023, PMID: 24406982

[ref62] Wiese H. Schweinberger S. R. (2011). Accessing semantic person knowledge: temporal dynamics of nonstrategic categorical and associative priming. J. Cogn. Neurosci. 23, 447–459. doi: 10.1162/jocn.2010.21432, PMID: 20146597

[ref63] Woldorff M. G. Fox P. T. Matzke M. Lancaster J. L. Veeraswamy S. Zamarripa F.. (1997). Retinotopic organization of early visual spatial attention effects as revealed by pet and ERPs. Hum. Brain Mapp. 5, 280–286. doi: 10.1002/(SICI)1097-0193(1997)5:4<280::AID-HBM13>3.0.CO;2-I, PMID: 20408229

[ref64] Wu K. Vassileva J. Noorian Z. Zhao Y. (2015). How do you feel when you see a list of prices? The interplay among price dispersion, perceived risk and initial trust in Chinese C2C market. J. Retail. Consum. Serv. 25, 36–46. doi: 10.1016/j.jretconser.2015.03.007

[ref65] Zhu Z. Jin Y. Su Y. Jia K. Lin C.-L. Liu X. (2022). Bibliometric-based evaluation of the neuromarketing research trend: 2010-2021. Front. Psychol. 13:872468. doi: 10.3389/fpsyg.2022.872468, PMID: 35983212 PMC9380815

